# Silencing ATF3 mediates mitochondrial homeostasis and improves ischemic stroke through regulating the MAPK signaling pathway

**DOI:** 10.3389/fnmol.2025.1554802

**Published:** 2025-06-20

**Authors:** Haifengqing Li, Fan Zhang, Cong Zhang, Min Zhou, Qing Liu, Guoyong Zeng

**Affiliations:** Department of Neurology, The Affiliated Ganzhou Hospital of Nanchang University, Ganzhou, China

**Keywords:** activating transcription factor 3, mitochondrial homeostasis, mitogen activated protein kinase, signaling pathway, ischemic stroke

## Abstract

Mitochondrial homeostasis is crucial for preventing and treatment of ischemic stroke. This study aimed to investigate the role of activating transcription factor 3 (ATF3) in ischemic stroke and mitochondrial homeostasis. ATF3 was silenced in oxygen glucose deprivation/reperfusion (OGD/R)-treated HT22 cells to evaluate its effects on cell apoptosis and mitochondrial function. The effects of silencing ATF3 on neurological injury, infarction, adenosine triphosphate (ATP), nicotinamide adenine dinucleotide (NAD+), mitofusin 1 (MFN1) and MFN2 were evaluated in stroke rats. Transcriptome sequencing and differential expression analysis were conducted to identify differential expressed genes (DEGs) associated with silencing ATF3, followed by functional enrichment analysis. The mitogen activated protein kinase (MAPK) agonist, anisomycin, was used to investigate the regulation of ATF3 in ischemic stroke and mitochondrial homeostasis via the MAPK pathway. Silencing ATF3 increased cell viability and inhibited apoptosis of OGD/R-induced cells. In stroke rats, silencing ATF3 reduced brain water content, decreased neurological injury and alleviated cerebral infarction. Notably, silencing ATF3 significantly inhibited the production of reactive oxygen species (ROS), increased the concentrations of ATP and NAD+, and upregulated the expression of MFN1 and MFN2. Next, 4,517 DGEs associated with silencing ATF3 were mainly enriched in MAPK signaling pathway. Silencing ATF3 downregulated the expression of phosphorylation-extracellular signal-regulated kinase (p-ERK)/ERK in OGD/R cells. Anisomycin notably reversed the effect of silencing ATF3 on ischemic stroke and mitochondrial homeostasis. Silencing ATF3 attenuates ischemic stroke and improves mitochondrial homeostasis via the MAPK signaling pathway, which shares a novel direction for maintaining mitochondrial homeostasis in ischemic stroke.

## Introduction

Ischemic stroke is one of the most common cerebrovascular events caused by blockage of a cerebral artery, and has been considered as a major contributor to mortality and morbidity worldwide, with the age-standardized incidence rate of 92.4/100,000 people and age-standardized mortality rate of 44.2/100,000 people (Li et al., 2024). Aging is a predominant non-modifiable risk of ischemic stroke, and the risk of ischemic stroke increases with population aging (Yousufuddin and Young, 2019). Additionally, the frequency of ischemic stroke occurrence varies among patients of different genders. The incidence of ischemic stroke is higher in men in early life, while ischemic stroke is more common in elderly women (Roy-O’Reilly and McCullough, 2018). Endovascular mechanical thrombectomy with or without intravenous thrombolysis are used treatments for ischemic stroke patients (Roaldsen et al., 2021). However, intravenous thrombolysis is suitable for use within 4.5 h after stroke onset, and the optimal time window for mechanical thrombectomy is only 24 h in carefully selected patients (Xiong et al., 2022). Therefore, development of new markers is urgent for ischemic stroke management.

Activating transcription factor 3 (ATF3), a stress-associated transcription factor, is a member of the ATF/cAMP response element-binding family. It has been reported that ATF3 can influence signal transduction and involves in cell death and differentiation (Liu et al., 2024). Typically, ATF3 is stably expressed under normal physiological conditions and highly expressed in response to injury or various stress such as oxidative damage and ischemia/reperfusion (I/R) (Rohini et al., 2018). Notably, the upregulated expression of serum ATF3 in oxygen–glucose deprivation/reoxygenation (OGD/R)-treated PC12 cells (Zheng et al., 2022). However, inhibition of ATF3 expression attenuates ischemic stroke progression through ferroptosis (Ye et al., 2022). Additionally, compelling evidence suggests that postprandial triglyceride-rich lipoproteins lipolysis products enhance mitochondrial reactive oxygen species (ROS) and promote ATF3 expression, resulting in cerebral microvascular endothelial cell damage, whereas inhibition of transcript variants 4 and variants 5 of ATF3 has been reported to decrease apoptosis and lipotoxicity in cerebral microvascular endothelial cells (Nyunt et al., 2019), indicating the relationship between ATF3 and cerebral mitochondrial function.

Mitochondrion is an organelle of cell and involves in adenosine triphosphate (ATP) energy production, homeostasis maintenance and communication with other organelles (Casanova et al., 2023). Recently, researchers have reported that mitochondria are frequently overlooked in the clinical management of ischemic stroke (Yang et al., 2018). The dysfunction of mitochondrion is recognized as one of the signs of neuron death caused by I/R, and keeping mitochondrial homeostasis plays a key role in reducing neuronal death and improving neurological function (Liu et al., 2018). Excessive mitochondrial ferritin alleviates blood–brain barrier damage after ischemic stroke by restoring endothelial cells iron homeostasis (Wang et al., 2022). Accumulating evidences have indicated the role of medicine in the prevention and treatment of ischemic stroke via maintaining mitochondrial function (Liu et al., 2023).

In this study, bioinformatics analysis was used to screen differential expressed genes (DEGs) between ischemic stroke rats and ischemic stroke rats with silencing ATF3, and to reveal the enriched signaling pathways for DEGs. Among the enriched signaling pathways, the mitogen activated protein kinase (MAPK) signaling pathway was selected as a potential regulatory pathway. The MAPK pathway is a pivotal pathway in eukaryotic signal transduction. Activation of the MAPK pathway followed by activation of its subfamilies including c-Jun amino-terminal kinase (JNK), extracellular signal-regulated kinase (ERK) and p38 via various stress results in neuronal cell death (Kumari et al., 2025). Meanwhile, enhanced p-p38 activity, neuronal apoptosis and mitochondrial damage after traumatic brain injury can be suppressed by p38 MAPK inhibitor (Yang et al., 2017), suggesting the important role of MAPK in cerebral injury. Hence, *in vivo* and *in vitro* experiments were conducted to investigate whether silencing ATF3 affected mitochondrial homeostasis and ischemic stroke development via the MAPK signaling pathway using the MAPK pathway agonist anisomycin.

## Methods

### Animals and ischemic stroke model

A total of 48 SD rats at 6–8 weeks (180-200 g) were bought from the SiPeiFu Biotechnology Co., Ltd. (Beijing, China). All rats were housed at a controlled condition (23 ± 2°C and 55–65% humidity) with free access for standard food and drinking water. Experiments were conducted according to the guidelines for laboratory animal care and approved by the local Ethics Committee.

One week after adaptively feeding, middle cerebral artery occlusion (MCAO) was induced in rats by injection of endothelin-1 (ET-1) to the middle cerebral artery. Rats were anesthetized with isoflurane (1.5%) and then placed in a prone position on a stereotactic frame. The skin of rats’ head was cut open along the midline of the peak to expose the bones, and a hole was drilled at anterior 0.9 mm and left 5.2 mm relative to bregma using a micro-hole drilling. A micro sampler was vertically inserted and 3uL of endothelin-1 (ET-1, 0.5 μg/μL, 600 pmol) (Park and Sohrabji, 2016) was injected at a rate of 1 μL/min when the depth reached 8.7 mm. Rats in sham group were injected with the same volume of saline into the brain. The needle was kept in position for 5 min to minimize backflow. The wound was sutured and rats were maintained between 36 and 37°C until woke up.

### Grouping and treatment

A total of 24 SD rats were randomly divided into sham group, MCAO group, lv-NC group and lv-ATF3 (*n* = 6 per group). Three weeks before induction of ischemic stroke model, rats in lv-NC group and lv-ATF3 group were injected with empty control and lentiviral particles knocking down ATF3 (2 × 10^9^ units/mL, OBiO Technology, Shanghai Corp., Ltd.) to the right lateral ventricle of rats. We slowly injected 10 μL of lentiviral particles 1.3 mm lateral (right) to the bregma and 1.5 mm to the back of the bregma using a 10-μL microliter syringe (Hamilton, #80300). The sequence of lv-ATF3 is TTCAACATCCAGGCCAGGTCT. Rats were subjected to neurological assessment and rotating rod test 24 h after ischemic stroke model induction.

Next, 24 SD rats were randomly divided into sham group, lv-NC group, lv-ATF3 group and lv-ATF3+anisomycin group (*n* = 6 per group). The MAPK pathway agonist, anisomycin (20 mg/kg) was intraperitoneally injected to rats 1 h before molding. All animal experiments underwent 3 biological replicates and at least 3 technical replicates. Rats were euthanized by excessive inhalation of isoflurane on the 4th day after MCAO and brain tissues were immediately collected for experiments.

### Brain water content detection

After euthanizing the rats, brain tissues were collected and immediately weighted using an analytical balance. Fresh brain tissues were dried at 105°C for 24 h, and the dried brain tissues were then weighted. The level of edema was evaluated using the formula: (wet weight-dry weight)/wet weight × 100%.

### Neurological assessment

Neurological assessment was conducted in ischemic stroke rats using the modified neurologic severity score (mNSS) (Min et al., 2017) before MCAO and 4, 7, 14, 21, 28, 35, 42 days after MCAO. The mNSS contains multiple neurological assessments including motor, reflex and balance tests. The mNSS was graded on a scale of 0–14 (normal score 0 to maximal deficit score 14). The higher the score was, the worse the sensorimotor function was.

### Rotating rod test

Ischemic stroke rats were placed on a 9 cm diameter rotating rod, which rotated at a speed of 30 rpm/min. Each rat was tested 3 times, and the interval of each test was 1 h. The rod rotator automatically recorded when each rat dropped its rod. The duration on the rod(s) was recorded until the rat fell from the rod. Rotating rod test was conducted before MCAO and after MCAO (4, 7, 14, 21, 28, 35, and 42 days) in each rat.

### 2,3,5,Triphenyl-2H-tetrazolium chloride (TTC) staining

TTC staining was conducted to evaluate the infarct area in brain tissue of rats. The intact brain tissues were obtained from SD rats and frozen at −20°C for 20 min for sectioning. The frozen intact brain tissue of rats was cut into 5 pieces every 2 mm. Next, slices were immersed in 2% TTC phosphate buffer solution (pH7.4) and incubated in a 37°C incubator for 30 min. After 15 min incubation, slices were flipped to ensure even contact with the staining solution. Slices were photographed and the degree of infarction was visualized. Infarct volume was analyzed using Image-Pro Plus (Media Cybernetics, USA).

### Cell culture

Mouse hippocampal neuron HT22 cells were bought from the iCell Bioscience Inc. (Shanghai, China). HT22 cells were maintained in DMEM medium containing 10% fetal bovine serum (FBS) and 100 U/mL penicillin/streptomycin at 37°C and 5% CO_2._ To simulate extracorporeal ischemic conditions, HT22 cells were cultured in an anoxic incubator containing 94% N_2_, 5% CO_2_ and 1% O_2_ at 37°C for 2 h (Alluri et al., 2015). Next, HT22 cells were incubated under normal conditions for 12 h. Cells not exposed to OGD/R were cultured for 12 h at 37°C with 5% CO_2_ and as a control. The MAPK pathway agonist anisomycin (0.2 μM) was added into HT22 cells for 48 h. Experiments performed in HT22 cells underwent 3 biological replicates and at least 3 technical replicates.

### Cell transfection

HT22 cells were divided into control group, si-NC group and si-ATF3 group. Twenty-four hours before cell transfection, HT22 cells (1 × 10^5^/well) were seeded into 6-well plates. Next, Lipofectamine 3,000 (ThermoFisher Scientific) was mixed with si-ATF3 according to the instructions, and the mixture was added to 6-well plates for 6 h. The medium containing siRNA-lipo3000 mixture was removed and replaced with fresh medium, and the 6-well plates were cultured in an incubator at 37°C and 5% CO_2_. The knockdown efficiency was assessed after transfection with si-ATF3-1, si-ATF3-2 and si-ATF3-3. The sequences for si-NC, si-ATF3-1, si-ATF3-2 and si-ATF3-3 were detailed in Supplementary Table 1.

### Cell viability

HT22 cells (1.5 × 10^4^cells/well) were seeded into 96-well plates and incubated for 24 h at 37°C and 5% CO_2_. After 24 h of cell culture, 10 μL of Cell Counting Kit-8 (CCK-8) reagent was added into cells and incubated in the dark for another 2 h. Finally, the absorbance at a wavelength of 450 nm was measured using a microplate reader.

### Cell apoptosis

Cell apoptosis was assessed using the Annexin V-FITC/propidium iodide (PI) staining. HT22 cells were digested using trypsin solution without EDTA and then centrifugated at 1000 g for 5 min. HT22 cells (3 × 10^5^cells/well) were treated with 5 μL of Annexin V and 10 μL of PI in the dark for 15 min at 20°C. Cell apoptosis was analyzed using Cytoflex flow cytometer (BD Biosciences, USA).

### Western blot analysis

RIPA lysis buffer (Biosharp Life Science, China) was added to cells and total proteins were obtained from cells and brain tissues. The Pierce BCA Protein Assay Kit (Thermo Fisher) was applied to detect protein concentration. The proteins were isolated using a 10% SDS-PAGE gel and then transferred to the PVDF membrane. After sealing with 5% skimmed milk, the membranes were incubated with the primary antibodies against ATF3 (1:1000, CST), B-cell lymphoma-2 (Bcl-2, 1:1000, CST), Bcl-2 associated X (BAX, 1:1000, CST), hypoxia-inducible factor 1 alpha (HIF-1α, 1:1000, CST), Caspase3 (1:1000, CST), phosphorylation-ERK (p-ERK, 1:2000, Proteintech), ERK (1:2000, Proteintech), mitofusin 1 (MFN1, 1:2000, Proteintech), MFN2 (1:5000, Proteintech) and aquaporin 4 (AQP4, 1:1000, CST) at 4°C overnight. Next, the membranes were then incubated with HRP-labeled secondary antibodies at room temperature for 1 h. Color was developed with ECL luminescence solution on Tanon 5,200 chemiluminescence imaging system. GAPDH was used as an internal control.

### Enzyme-linked immunosorbent assay (ELISA)

The levels of malondialdehyde (MDA), superoxide dismutase (SOD), reactive oxygen species (ROS), lactate dehydrogenase (LDH), adenosine triphosphate (ATP), and nicotinamide adenine dinucleotide (NAD+) in the brain of rats were determined using ELISA kits according to the manufactures’ instructions.

### Identification of differentially expressed genes (DEGs)

Three brain tissues of MCAO rats and MCAO+lv-ATF3 rats were selected for transcriptome sequencing, and DESeq2^1^ was used to perform differential expression analysis between MCAO+lv-ATF3 group and MCAO group. DEGs were screened under the threshold of |log2FoldChange| > 1 and *p* value < 0.05. Volcano plots of DEGs were drawn by using ggplots2 R package. The pheatmap R package was used to conduct bidirectional cluster analysis for DEGs. Distance was calculated using Euclidean method and hierarchical clustering was performed by Complete Linkage method.

### Enrichment analysis for DEGs

DAVID,^2^ the online functional annotation tool, was applied for enrichment analysis of shared DEGs. Gene Ontology (GO) enrichment analyses included biological process (BP), molecular function (MF) and cellular component (CC) categories. The potential biological pathways were identified by Kyoto Encyclopedia of Genes and Genomes (KEGG) enrichment analysis. Significant pathways were displayed with bubble charts.

### Statistical analysis

All experimental data was processed by GraphPad Prism 8.0 software (GraphPad Prism Software Inc., San Diego, CA, USA) and presented in the form of means ± standard deviation (SD). T-tests were used for comparisons between two groups. One-way analysis of variance (ANOVA) was used for comparisons among multiple groups, followed by Tukey’s *post hoc* test. *p* < 0.05 indicated that the difference was statistically significant.

## Results

### Silencing ATF3 promotes OGD/R-induced HT22 cell viability and inhibits cell apoptosis

**Figure 1:**
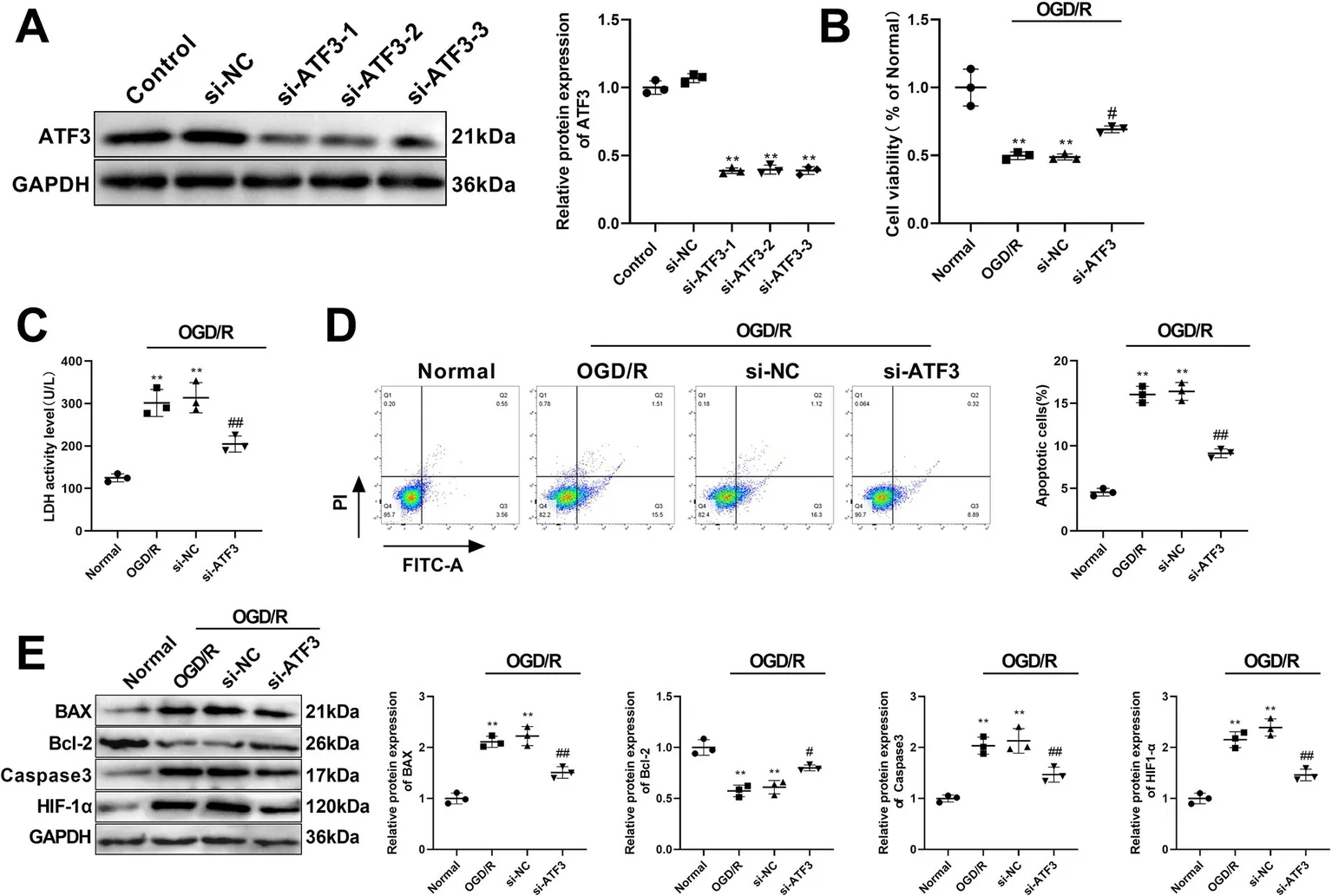
Silencing ATF3 promotes cell proliferation and inhibits cell apoptosis in OGD/R-treated HT22. **(A)** Western blot analysis assesses silencing efficiency of si-ATF3-1, si-ATF3-2 and si-ATF3-3 in HT22 cells. **(B)** CCK-8 assesses cell viability of OGD/R-treated HT22 cells after transfection with si-ATF3. **(C)** ELISA measures the level of LDH activity in OGD/R-treated HT22 cells after transfection with si-ATF3. **(D)** Flow cytometry analyzes cell apoptosis of OGD/R-treated HT22 cells after transfection with si-ATF3. **(E)** Western blot analysis determines the protein levels of BAX, Bcl-2, Caspase3 and HIF-1α in OGD/R-treated HT22 cells after silencing ATF3. ^**^p < 0.01, vs. normal group. ^#^*p* < 0.05 and ^##^*p* < 0.01, vs. OGD/R or si-NC group.

ATF3 is silenced in HT22 cells and the knockdown efficiency of ATF3 was verified using western blot analysis. As shown in Figure 1A, the relative protein expression of ATF3 was significantly downregulated after transfection with si-ATF3-1, si-ATF3-2 and si-ATF3-3 compared with control cells (*p* < 0.01). Next, si-ATF3-3 was selected for further experiments. The effect of si-ATF3 on cell viability of OGD/R-treated HT22 cells was assessed. OGD/R induced lower cell viability than that of normal cells (*p* < 0.01), while transfection with si-ATF3 considerably increased cell viability of OGD/R-induced HT22 cells compared with OGD/R or si-NC cells (*p* < 0.05) (Figure 1B). Additionally, transfection with si-ATF3 notably decreased higher LDH activity level caused by OGD/R (*p* < 0.01) (Figure 1C). OGD/R significantly increased the apoptotic cell rate compared with normal cells (*p* < 0.01), whereas silencing ATF3 suppressed OGD/R-induced cell apoptosis (*p* < 0.01) (Figure 1D). Also, we determined the expression levels of HIF-1α, Caspase3 and apoptosis-related proteins including BAX and Bcl-2 after silencing ATF3. The results displayed that the levels of BAX, HIF-1α and Caspase3 were significantly higher and Bcl-2 expression was significantly lower in OGD/R and si-NC cells than normal cells (*p* < 0.01), while silencing ATF3 dramatically downregulated BAX (*p* < 0.01), HIF-1α (*p* < 0.01) and Caspase3 (*p* < 0.01) and upregulated Bcl-2 expression (*p* < 0.05) compared with OGD/R or si-NC cells (Figure 1E). Collectively, silencing ATF3 could promote cell viability and inhibit cell apoptosis in OGD/R-induced HT22 cells.

### Silencing ATF3 improves mitochondrial function in OGD/R-treated HT22 cells

**Figure 2:**
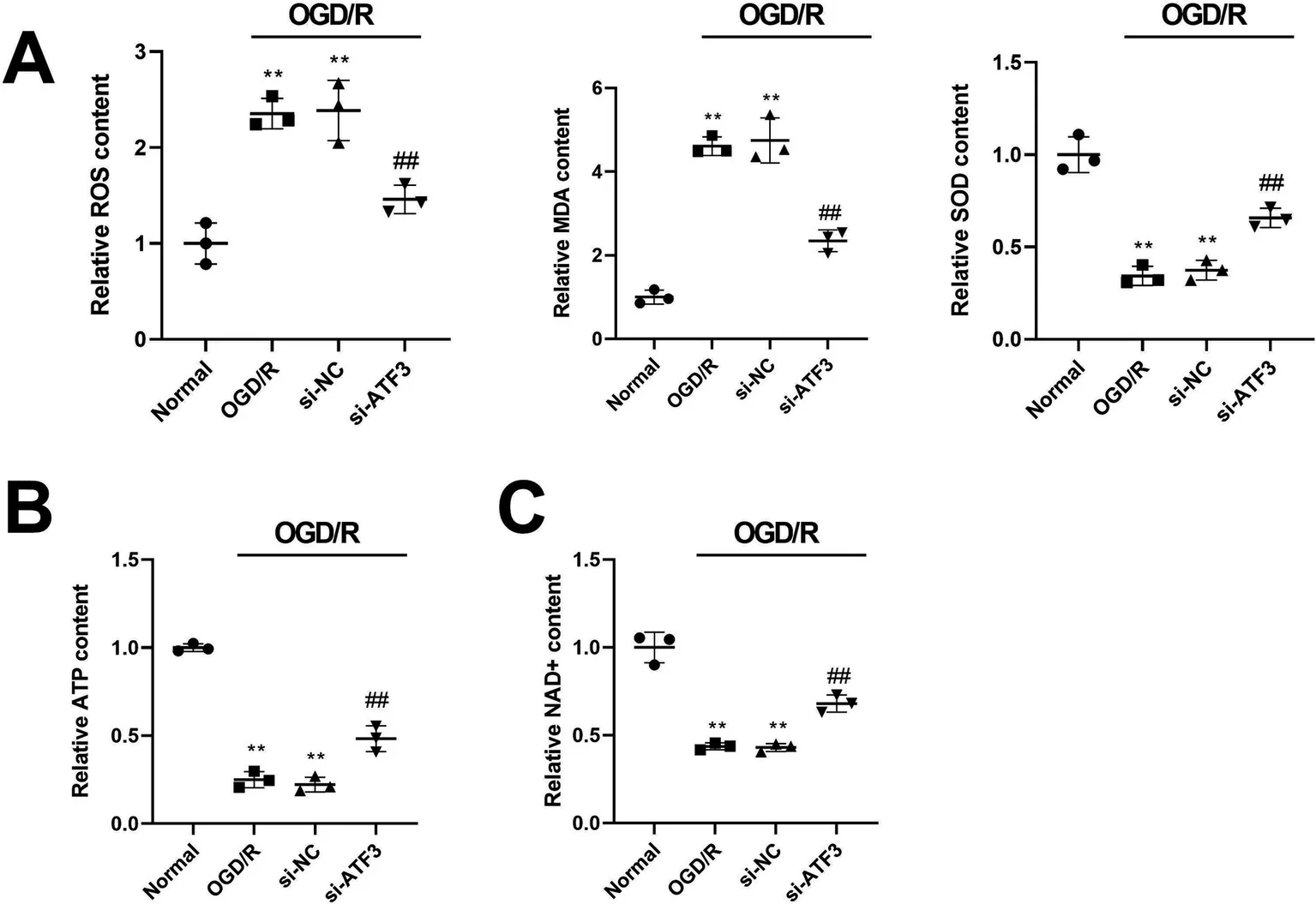
Silencing ATF3 expression improves mitochondrial function in OGD/R-treated HT22 cells. **(A)** ELISA measures the production of ROS, MDA, and SOD in OGD/R-treated HT22 cells after silencing ATF3. **(B,C)** ELISA detects the concentrations of mitochondrial functional markers ATP and NAD + in OGD/R-treated HT22 cells after silencing ATF3. ^**^p < 0.01, vs. normal group. ^##^*p* < 0.01, vs. OGD/R or si-NC group.

The concentrations of oxidative stress markers such as ROS, MDA and SOD were detected by ELISA. The contents of ROS and MDA were significantly increased, and the content of SOD was decreased induced by OGD/R compared with normal cells (*p* < 0.01); silencing ATF3 decreased the contents of ROS and MDA and increased the content of ROS compared with OGD/R or si-NC cells (*p* < 0.01) (Figure 2A), indicating the inhibitory effect of silencing ATF3 on oxidative stress in OGD/R-treated HT22 cells. Meanwhile, we measured the effects of silencing ATF3 on mitochondrial function. The concentrations of mitochondrial functional markers such as ATP and NAD+ were measured using ELISA, and the results showed that the concentrations of ATP and NAD+ in OGD/R or si-NC cells were significantly reduced compared with normal cells (*p* < 0.01), whereas transfection with si-ATF3 considerably increased the concentrations of ATP and NAD+ compared with OGD/R or si-NC cells (*p* < 0.01) (Figures 2B,C). The findings suggested that silencing ATF3 alleviated OGD/R-induced mitochondrial dysfunction in HT22 cells.

### Silencing ATF3 alleviates ischemic stroke progression and regulates mitochondrial homeostasis in rats

**Figure 3:**
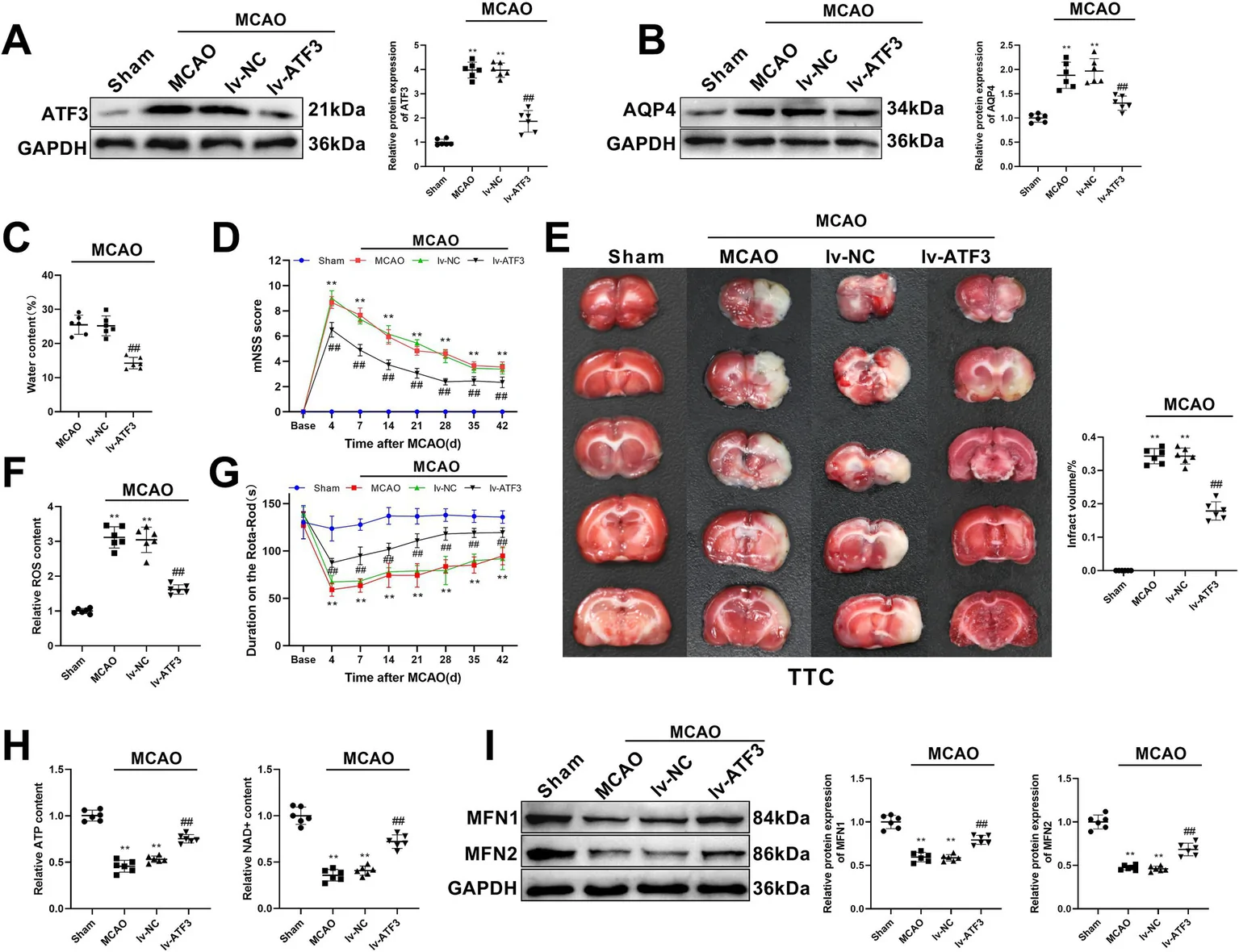
Silencing ATF3 alleviates ischemic stroke progression and regulates mitochondrial homeostasis in rats. **(A)** Western blot determines the expression of ATF3 in the brain of ischemic stroke rats after silencing ATF3. **(B)** Western blot determines the expression of AQP4 in the brain of ischemic stroke rats after silencing ATF3. **(C)** Brain water content of ischemic stroke rats after injection with lv-ATF3. **(D)** Neurological assessment (mNSS score) of rats before MCAO and after MCAO (4, 7, 14, 21, 28, 35 and 42 days). **(E)** TTC staining displays the degree of infarction of the brain in MCAO rats and lv-ATF3 rats. Scatter plots are generated to show infarct volume (%) of rats. **(F)** ELISA detects the content of ROS in the brain of rats after injection with lv-ATF3. **(G)** Duration on the rota-rod of rats before MCAO and after MCAO (4, 7, 14, 21, 28, 35 and 42 days). **(H)** ELISA measures the production of ATP and NAD + in the brain of ischemic stroke rats after silencing ATF3. **(I)** Western blot analysis determines the expression of MFN1 and MFN2 after silencing ATF3. ^**^p < 0.01, vs. sham group. ^#^*p* < 0.05 and ^##^*p* < 0.01, vs. MCAO or lv-NC group.

Furthermore, ATF3 expression was silenced by injection with lv-ATF3 in ischemic stroke rats. The expression of ATF3 was significantly upregulated in MCAO rats compared with sham rats (*p* < 0.01), whereas injection with lv-ATF3 considerably inhibited the expression of ATF3 compared with MCAO or lv-NC rats (*p* < 0.01) (Figure 3A). Ischemic stroke rats exhibited a higher expression level of AQP4 than that of sham rats (*p* < 0.01), while silencing ATF3 in MCAO rats significantly downregulated the expression of AQP4 compared with MCAO or lv-NC rats (*p* < 0.01) (Figure 3B). Additionally, MCAO rats had increased water content in the brain, and silencing ATF3 notably decreased water content compared with MCAO or lv-NC rats (*p* < 0.01) (Figure 3C). Neurological assessment and rotating rod test were conducted on ischemic stroke rats. The results showed that the mNSS score of rats was highest after 4 days of MCAO, indicating that nerve damage was most severe after 4 days of MCAO. Rats in MCAO and lv-NC groups had significant increased mNSS score compared with sham rats (*p* < 0.01), whereas rats in lv-ATF3 group exhibited a significant reduction in mNSS score compared with MCAO and lv-NC rats (*p* < 0.01) (Figure 3D). TTC staining displayed that MCAO or lv-NC rats had notable infarct area compared with sham rats (*p* < 0.01), while silencing ATF3 alleviated infarction of brain tissue compared with MCAO or lv-NC rats (*p* < 0.01) (Figure 3E). The production of ROS was higher in MCAO group than that of sham group (*p* < 0.01), while silencing ATF3 significantly suppressed the content of ROS in the brain of rats compared with MCAO or lv-NC group (*p* < 0.01) (Figure 3F). The shortest duration on the rota-rod was observed on the 4th day after MCAO. Rats in MCAO group and lv-NC group had decreased rota-rod duration compared with sham rats (*p* < 0.01), while rats in lv-ATF3 group exhibited a significant increase in duration on the rota-rod compared with MCAO and lv-NC rats (*p* < 0.01) (Figure 3G), which indicated that silencing ATF3 could improve the motor coordination of stroke rats. Next, we evaluated the levels of mitochondrial homeostasis markers ATP and NAD+, and the results revealed that silencing ATF3 dramatically promoted the production of ATP and NAD+ (*p* < 0.01) (Figure 3H). Mitochondrial homeostasis-related proteins such as MFN1 and MFN2 were quantified by western blot analysis. Silencing ATF3 induced higher protein levels of MFN1 (*p* < 0.05) and MFN2 (*p* < 0.01) than that of MCAO or lv-NC (Figure 3I). The results indicated that silencing ATF3 could alleviate ischemic stroke development and improve mitochondrial homeostasis in rats.

### Identification of DEGs and functional enrichment analyses

Three brain tissue samples from MCAO and MCAO+lv-ATF3 rats were subjected for transcriptome sequencing. A total of 4,517 DGEs were found between MCAO group and MCAO+lv-ATF3 group, which comprised 1,285 upregulated genes and 3,232 downregulated genes (Supplementary Figure 1A). Supplementary Figure 1B displayed the expression patterns of DGEs. The top 15 upregulated genes and top 15 downregulated genes were detailed in Supplementary Table 2.

**Figure 4:**
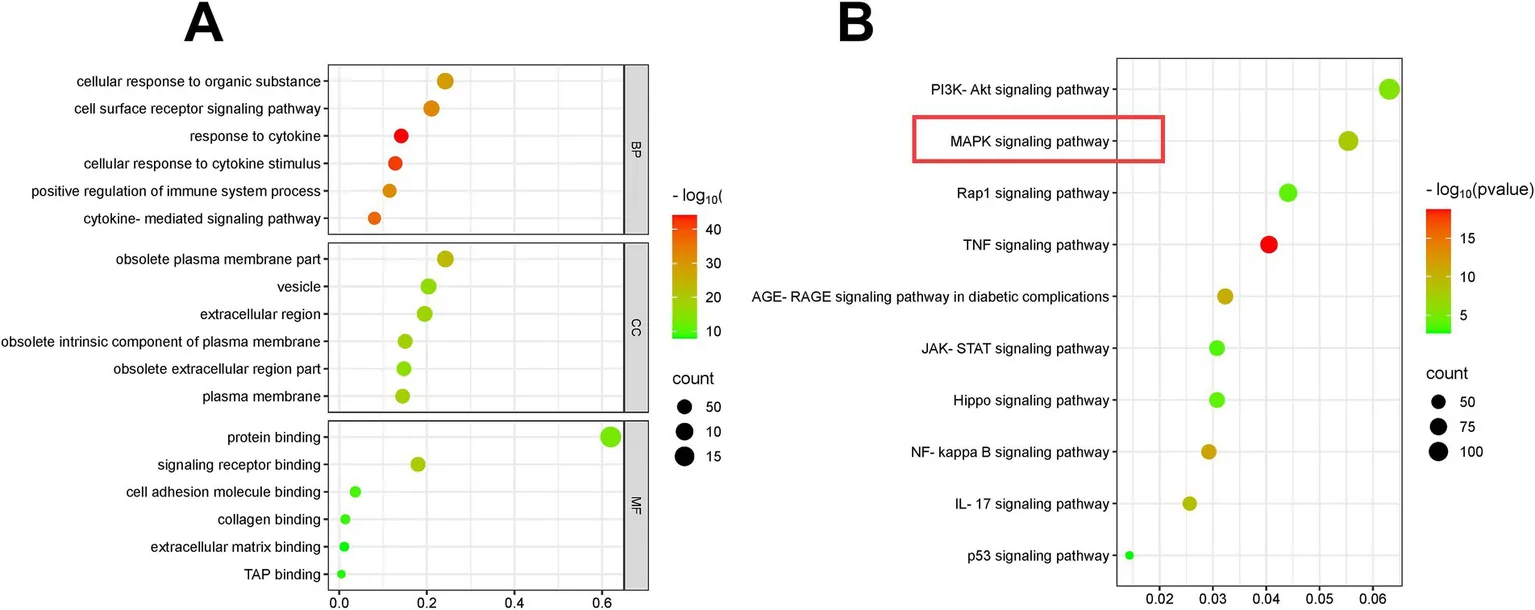
GO and KEGG enrichment analyses for DEGs. **(A)** Bubble diagram of GO enrichment analysis. The top 6 enriched items of BP category, CC category, and MF category are displayed. **(B)** The top 10 enriched KEGG pathways.

Subsequently, 4,517 DGEs were selected for GO and KEGG enrichment analyses. In BP category, these DGEs were mainly enriched in cellular response to organic substance, cell surface receptor signaling pathway, response to cytokine, cellular response to cytokine stimulus, positive regulation of immune system process, and cytokine-mediated signaling pathway; In CC category, DGEs were predominantly abundant in obsolete plasma membrane part, vesicle, extracellular region, obsolete intrinsic component of plasma membrane, obsolete extracellular region part, and plasma membrane; In MF category, protein binding, signaling receptor binding, cell adhesion molecule binding, collagen binding, extracellular matrix binding, and TAP binding were the top 6 enriched items (Figure 4A). KEGG analysis revealed that DEGs were mainly abundant in the PI3K-Akt signaling pathway, MAPK signaling pathway, Rap1 signaling pathway, and TNF signaling pathway (Figure 4B).

### ATF3 affects ischemic stroke and regulates mitochondrial homeostasis via the MAPK pathway in HT22 cells

**Figure 5:**
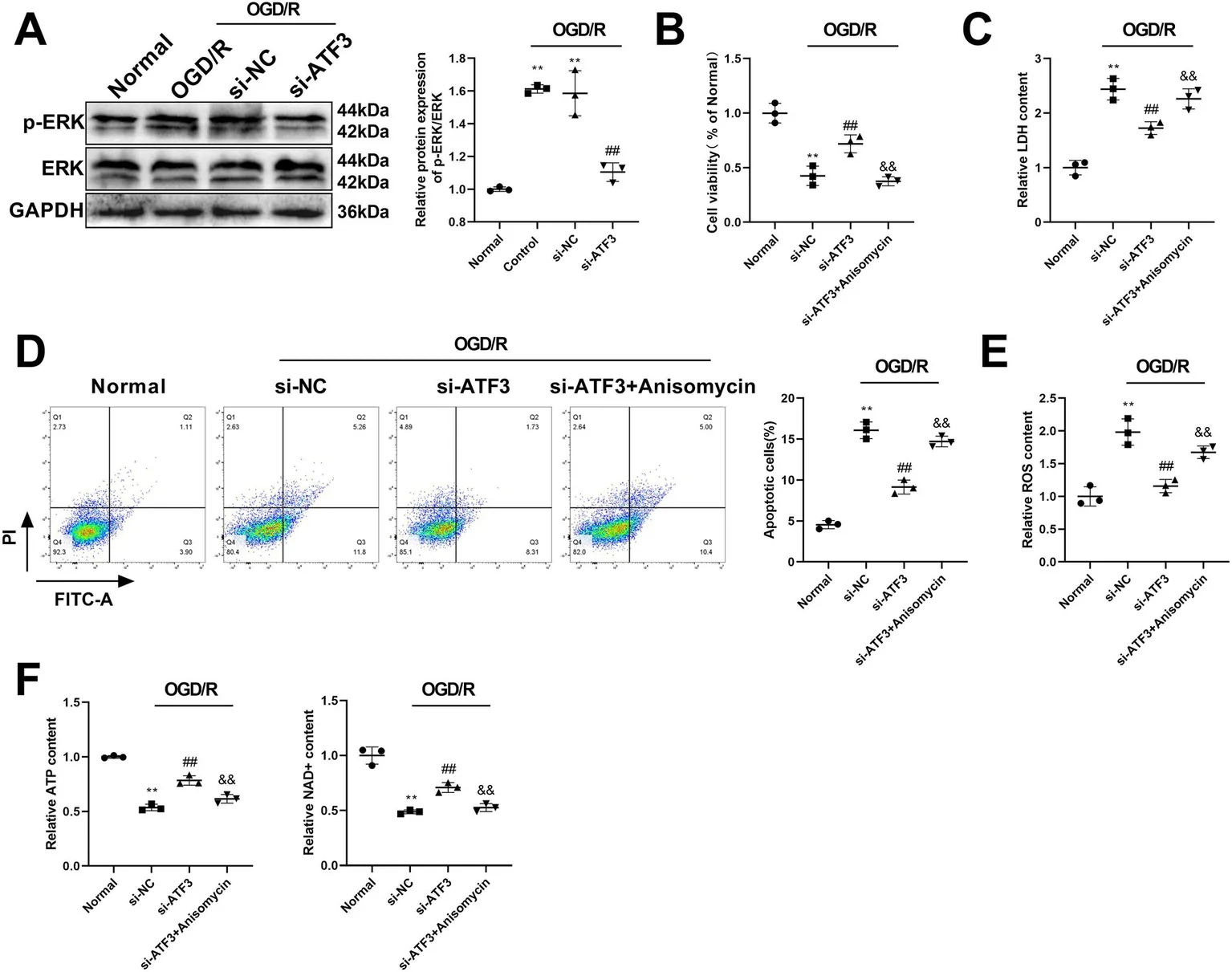
ATF3 affects ischemic stroke and regulates mitochondrial homeostasis via the MAPK pathway in HT22 cells. **(A)** The expression of p-ERK/ERK is detected by western blot analysis after transfection with si-ATF3 in OGD/R-treated cells. **(B)** Cell viability is assessed by CCK-8 in si-ATF3-treated HT22 cells after adding anisomycin. **(C)** The relative LDH content is measured by ELISA in si-ATF3-treated HT22 cells after adding anisomycin. **(D)** Flow cytometry analyzes cell apoptosis of si-ATF3-treated HT22 cells after adding anisomycin. **(E)** ELISA measures the content of ROS in si-ATF3-treated HT22 cells after using anisomycin. **(F)** ELISA detects the concentrations of ATP and NAD + in si-ATF3-treated HT22 cells after using anisomycin. ^**^p < 0.01, vs. normal group. ^##^*p* < 0.01, vs. si-NC group. ^&&^*p* < 0.01, vs. si-ATF3 group.

To investigate the effect of ATF3 on the MAPK pathway, we determined the expression levels of key proteins in the MAPK pathway through western blot analysis. Figure 5A showed that OGD/R significantly promoted the expression of p-ERK/ERK compared with normal cells (*p* < 0.01), while silencing ATF3 in OGD/R cells considerably suppressed the expression of p-ERK/ERK compared with si-NC cells (*p* < 0.01). Furthermore, anisomycin, the MAPK pathway agonist, was added into si-ATF3 cells. As shown in Figure 5B, silencing ATF3 increased cell viability of OGD/R cells compared with si-NC cells (*p* < 0.01), and application of anisomycin significantly inhibited cell viability compared with si-ATF3 cells (*p* < 0.01). Meanwhile, application of anisomycin significantly induced a higher content of LDH than that of si-ATF3 cells (*p* < 0.01) (Figure 5C). Silencing ATF3 notably suppressed the apoptosis rate of HT22 cells, whereas, anisomycin significantly increased cell apoptosis compared with si-ATF3 cells (*p* < 0.01) (Figure 5D). ELISA was used to detect the concentrations of oxidative stress markers and mitochondrial homeostasis markers. The results revealed that the ROS concentration was significantly decreased and the concentrations of ATP and NAD + were significantly increased in si-ATF3 cells compared with si-NC cells (*p* < 0.01). However, anisomycin induced a significant increase in ROS concentration and a significant decrease in ATP and NAD + concentration compared with si-ATF3 cells (*p* < 0.01) (Figures 5E,F). The findings suggested that silencing ATF3 might inhibit apoptosis in OGD/R-treated HT22 cells and improve mitochondrial homeostasis through the MAPK pathway.

### ATF3 affects ischemic stroke development and improves mitochondrial homeostasis through the MAPK pathway in rats

**Figure 6:**
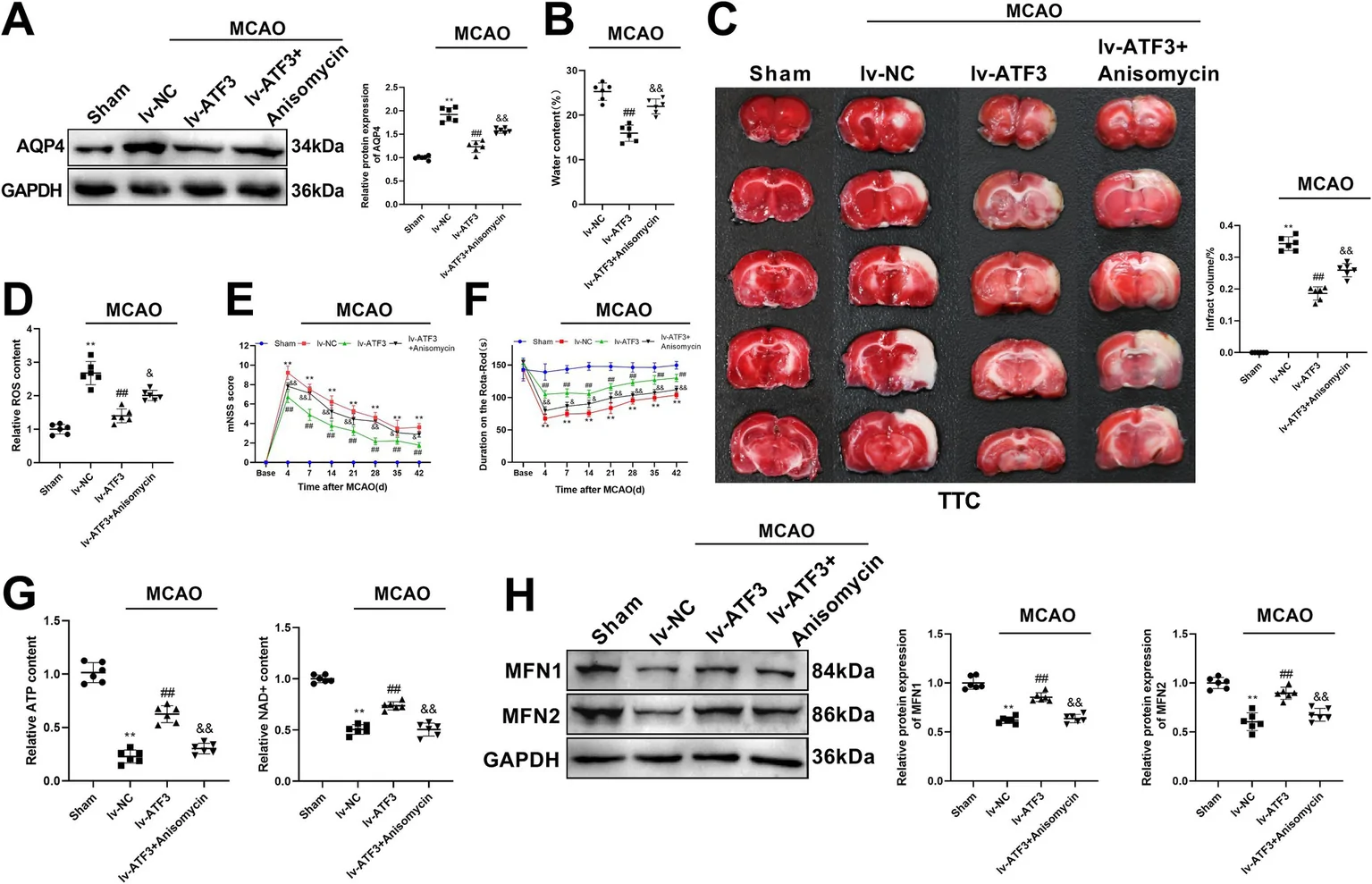
ATF3 affects ischemic stroke development and improves mitochondrial homeostasis through the MAPK pathway in rats. **(A)** Western blot determines the expression of AQP4 in lv-ATF3 rats after administration of anisomycin. **(B)** Brain water content of lv-ATF3 rats after administration of anisomycin. **(C)** TTC staining displays the degree of infarction of the brain in lv-ATF3 rats after using anisomycin. Scatter plots are generated to show infarct volume (%) of rats. **(D)** ELISA detects the content of ROS in the brain of lv-ATF3 rats after using anisomycin. **(E)** Neurological assessment (mNSS score) before MCAO and after MCAO (4, 7, 14, 21, 28, 35 and 42 days) in lv-ATF3 + anisomycin group. **(F)** Duration on the rota-rod before MCAO and after MCAO (4, 7, 14, 21, 28, 35 and 42 days) in lv-ATF3 + anisomycin group. **(G)** ELISA measures the levels of ATP and NAD + in the brain of lv-ATF3 rats after using anisomycin. **(H)** Western blot determines the levels of MFN1 and MFN2 in the brain of lv-ATF3 rats after using anisomycin. ^**^p < 0.01, vs. sham group. ^##^*p* < 0.01, vs. lv-NC group. ^&^*p* < 0.05 and ^&&^*p* < 0.01, vs. lv-ATF3 group.

The MAPK pathway agonist anisomycin was further administrated in ischemic stroke rats, in order to investigate the role of ATF3 in ischemic stroke and mitochondrial homeostasis through the MAPK pathway. Silencing ATF3 significantly decreased the expression of AQP4 and reduced water content in the brain of ischemic stroke rats compared with lv-NC group (*p* < 0.01), whereas the inhibitory of silencing ATF3 was weakened by anisomycin (*p* < 0.01) (Figures 6A,B). Cerebral infarction of rats was alleviated by lv-ATF3 compared with lv-NC rats (*p* < 0.01), but administration of anisomycin significantly increased infarct area compared with lv-ATF3 rats (*p* < 0.01) (Figure 6C). In addition, administration of anisomycin in lv-ATF3 rats weakened the inhibitory effect of lv-ATF3 on ROS concentration (*p* < 0.05) (Figure 6D). The mNSS score was highest on the 4th day after MCAO and gradually decreased over time. Silencing ATF3 notably decreased mNSS score compared with lv-NC group (*p* < 0.01), while administration of anisomycin in lv-ATF3 rats increased mNSS score compared with lv-ATF3 rats (*p* < 0.05 or *p* < 0.01) (Figure 6E). MCAO rats had a shorter duration on the rota-rod on the 4th day after MCAO than other time points. Silencing ATF3 significantly increased duration on the rota-rod compared with lv-NC rats (*p* < 0.01), whereas administration of anisomycin notably decreased duration on the rota-rod compared with lv-ATF3 rats (*p* < 0.05 or *p* < 0.01) (Figure 6F). Meanwhile, anisomycin suppressed the concentrations of ATP and NAD + compared with lv-ATF3 group (*p* < 0.01) (Figure 6G). The expression of mitochondrial homeostasis-related proteins MFN1 and MFN2 was detected by western blot analysis, and the results showed that the expression levels of MFN1 and MFN2 in the brain of lv-ATF3 rats were significantly elevated compared with lv-NC rats (*p* < 0.01), whereas administration of anisomycin significantly suppressed their expression compared with lv-ATF3 group (*p* < 0.01) (Figure 6H). Collectively, silencing ATF3 attenuated the progression of ischemic stroke and improved mitochondrial homeostasis through regulating the MAPK pathway.

## Discussion

Ischemic stroke remains a cerebrovascular event with high rates of death and disability with limited treatment approaches. In recent years, novel therapeutic targets for ischemic stroke have been investigated by researchers, in order to improve cerebral injury after ischemic stroke (Liu et al., 2022; Shen et al., 2022). In the present study, ATF3 is silenced in OGD/R-induced HT22 cells and ischemic stroke rats, and we found silencing ATF3 could alleviate ischemic stroke and improve mitochondrial homeostasis. Furthermore, DEGs associated with silencing ATF3 were identified between MCAO and MCAO+lv-ATF3 rats and they were mainly enriched in the MAPK signaling pathway. Next, silencing ATF3 considerably suppressed the expression of p-ERK/ERK, and anisomycin, the MAPK pathway agonist, reversed the effects of silencing ATF3 on ischemic stroke and mitochondrial homeostasis, indicating that silencing ATF3 attenuated ischemic stroke and regulated mitochondrial homeostasis via the MAPK signaling pathway.

In Kao et al. (2023) ATF3 expression was rapidly and significantly induced 0.5 h after the onset of MCAO, and ATF3 expression continued to rise during reperfusion, peaking at 12 h and then gradually declining. The results showed that ATF3 knockout (KO) mice exhibited larger infarct area than wild type (WT) mice after 25 min of MCAO and 24 h of reperfusion. Next, the replication-defective recombinant adenoviral (rAd) vector was used to construct a human phosphoglycerate kinase (hPGK) promoter to drive ATF3 (Ad-ATF3), and infarct volume in KO mice receiving Ad-ATF3 was notably reduced compared with Ad-hPGK control mice. The results from Kao were inconsistent with our results, and several factors might contribute to this discrepancy: (1), Kao found a transient expression of ATF3 in MCAO mice, and the genome of adenovirus was not be integrated into the host cell genome, therefore it could not be used to construct stable cell lines and is only suitable for transient expression. In this study, we conducted lentiviral plasmid injection 3 weeks prior to MCAO model for long-term knockdown of gene expression. Therefore, transient transfection and long-term stable transfection might have an impact on the results. (2), in the present study, silencing ATF3 gene did not alter the DNA sequence and it only causes a decrease in gene function or partial loss of function without stopping gene expression; in Kao et al. (2023), gene knockout was used in mice, which rendered specific genes in an organism inactive. The impact of these two technologies on ATF3 gene expression was different, which might have different effects on the results. (3), we used ET-1 to construct a stroke model, and MCAO rats had the highest mNSS score on the the 4th day after MCAO. We collected samples on the 4th day and then measured the infarct area. However, Kao et al. (2023) only examined the infarct size after 25 min of MCAO and 24 h of reperfusion. Different processing points might also be a reason for inconsistency. Furthermore, our previous study has identified that ATF3 is upregulated in ischemic stroke rats through transcriptome sequencing, and knockdown of ATF3 alleviates ischemic stroke by inhibiting ferroptosis after knocking down ATF3 in ischemic stroke rats (Ye et al., 2022). Therefore, this study aimed to silence ATF3 to investigate its downstream mechanism on ischemic stroke.

NAD+/NADH is a redox pair in cells, where NADH is the reduced form of NAD and NAD+ is its oxidized form. NAD+ is crucial in various biological processes such as energy metabolism and mitochondrial functions. Ischemia induces succinate accumulation and mitochondrial complex I activity deficient, which promotes reverse electron transfer. During this process, a part of the electrons from succinate are redirected to the NADH dehydrogenase module of complex I, where the substrate NAD+ is converted to NADH and thereby producing excess ROS (Chavda and Lu, 2023). Inhibition of mitochondrial complex I may cause the imbalance of NAD+/NADH redox state (Gao et al., 2022), whereas restoration of NAD+/NADH balance improves mitochondrial respiration and reduces mitochondrial oxidant production (Titov et al., 2016). Notably, application of NAD+ suppresses oxidative cell death and ischemic brain injury (Ma et al., 2015). In hyperglycemia-induced MCAO rats, decreased levels of ATP and NAD + are observed, whereas ATP content and NAD+ content are elevated, and the infarction is inhibited after exposure to hyperbaric oxygen (Hu et al., 2017), which indicate the protective role of NAD+ in ischemic stroke. Wang and colleagues have revealed that supplementation with NAD+ and butylphthalide significantly reduces the production of MDA and ROS, as well as alleviates mitochondrial injury after ischemic stroke in mice (Wang et al., 2023), which suggests that increased NAD+ content may be benefit for ischemic stroke and mitochondrial homeostasis. In this study, the levels of ATP and NAD + were both downregulated after induction of ischemic stroke, while silencing ATF3 significantly upregulated the expression of ATP and NAD+. The findings might indicate silencing ATF3 restore mitochondrial energy homeostasis by increasing NAD+.

After occurrence of stroke, mitochondrial fission and fusion are closely related to mitochondrial integrity and neurological damage. MFN1 and MFN2 are two mitochondrial transmembrane proteins and play crucial role in mitochondrial fusion. Also, MFN2 has been recognized as a key regulator of cellular energy and mitochondrial metabolism (Emery and Ortiz, 2021). However, dysfunction of MFN1 and MFN2 is associated with pathological conditions. Decreased expression of MFN1 and MFN2 in OGD-treated neuronal cells and MCAO rats causes Ca^2+^ overload and translocation of BAX to mitochondria (Yang et al., 2022). Huang and colleagues have found that application of ginsenoside compound K after I/R inhibits the binding affinity of Mul1 and MFN2, and therefore suppresses MFN2 ubiquitination and degradation, leading to increased expression of MFN2, indicating an important role of MFN2 in mitochondrial homeostasis (Huang et al., 2023). Gao et al. (2019) has revealed that administration of resveratrol can regulate mitochondrial homeostasis by activating the MFN1-associated mitochondrial protective system after I/R injury, while inhibition of MFN1 weakened the effect of resveratrol on mitochondrial homeostasis. Additionally, salvinorin A attenuates focal cerebral ischemic injury and preserves mitochondrial morphology and functions via increasing the level of MFN2 and activating AMPK (Dong et al., 2019). In consistent with previous studies, the present study showed that the expression levels of MFN1 and MFN2 were significantly elevated after silencing ATF3 in OGD/R-treated cells and ischemic stroke rats, suggesting the protective effect of silencing ATF3 on mitochondrial homeostasis.

Previous studies have revealed the implication of ERK1/2 in ischemic stroke, however, the increased phosphorylation of ERK1/2 is protective or harmful for ischemic stroke is still controversial (Sawe et al., 2008). Some pathogenic factors such as cytokines, free radicals and inflammation promoting ERK1/2 activity may aggravate cerebral ischemic injury, while other factors including exogenous growth factors, estrogen or preconditioning inducing ERK1/2 activity may protective after ischemia (Sawe et al., 2008). Wu has found that electroacupuncture attenuates cerebral injury after stroke through inhibiting apoptosis and increasing the level of p-ERK pathway (Wu et al., 2015). On the contrary, application of myricetin after ischemic stroke notably suppresses apoptosis caused by oxidative stress and inflammation, accompanied with decreased expression of p-ERK (Zhang et al., 2023). Herein, we observed that the expression of p-ERK/ERK was increased in OGD/R cells, whereas silencing ATF3 in OGD/R cells considerably suppressed the expression of p-ERK/ERK. Furthermore, administration of anisomycin reversed the results. This study has discovered for the first time that silencing ATF3 could alleviate ischemic stroke and improve mitochondrial homeostasis via regulating the MAPK pathway.

There may still be some limitations that need to be noticed. In this study, we have investigated the role of silencing ATF3 in mitochondrial homeostasis, ischemic stroke progression and the MAPK signaling pathway in the *in vivo* and *in vitro* experiments, however, the experiments simulated what happened in animals and cells, but the tissue or cells were still different from those in humans. Hence, the effect of silencing ATF3 on ischemic stroke should be validated by extensive representative studies. The MAPK signaling pathway was one of the enriched pathways of DEGs, and other pathways such as PI3K-Akt signaling pathway and Rap1 signaling pathway were significantly enriched. Therefore, the role of ATF3 in these pathways can also be further investigated, in order to order to complement the potential mechanisms of action for ATF3 in ischemic stroke. We injected lentiviral particles into rats 3 weeks before ischemic stroke model induction, and the results showed good silencing efficiency of si-ATF3, which indicating that lentiviral particles integrated into the host genome could persistently inhibit the expression of target genes. Transient inhibition should be investigated in subsequent studies. The effect of ATF3 overexpression is worthy studying in the future to provide important insights into its dual roles and potential mechanistic pathway. Laser doppler flowmetry or magnetic resonance imaging is a useful tool to study the cerebral blood flow of MCAO rats, unfortunately, we did not have this necessary device to study cerebral blood flow. Additionally, studying the effect of silencing ATF3 on mitochondrial morphology would be useful to understand the details of silencing ATF3 on ischemic stroke. We hope, in the future, to employ techniques to determine these issues.

In conclusion, the results demonstrated that silencing ATF3 improved mitochondrial homeostasis and inhibited ischemic stroke progression through inactivating the MAPK signaling pathway. This study suggested ATF3 as a promising marker for ischemic stroke and revealed the MAPK signaling pathway as the potential regulatory pathway of ATF3 in ischemic stroke.

## Data Availability

The data presented in the study are deposited in the SRA repository (https://www.ncbi.nlm.nih.gov/sra/PRJNA1272262), accession number PRJNA1272262.

## Footnotes

1
https://bioconductor.org/packages/release/bioc/html/DESeq2.html

2
https://david.ncifcrf.gov/summary.jsp
