# Correction: Silencing ATF3 mediates mitochondrial homeostasis and improves ischemic stroke through regulating the MAPK signaling pathway

**DOI:** 10.3389/fnmol.2026.1940362

**Published:** 2026-08-07

**Authors:** Haifengqing Li, Fan Zhang, Cong Zhang, Min Zhou, Qing Liu, Guoyong Zeng

**Affiliations:** Department of Neurology, The Affiliated Ganzhou Hospital of Nanchang University, Ganzhou, China

**Keywords:** activating transcription factor 3, mitochondrial homeostasis, mitogen activated protein kinase, signaling pathway, ischemic stroke

In the published article, an incorrect **Funding** statement was provided. The second funder was incorrectly included as “Natural Science Foundation of Jiangxi Province (S2023ZRMSL0428)”. The corrected **Funding** statement appears below.

“The author(s) declared that financial support was received for this work and/or its publication. This study was supported by the National Natural Science Foundation of China (82360251) and Jiangxi Provincial Natural Science Foundation (20232BAB206067).”

The original version of this article has been updated.

