# Supplementary material for: Silencing ATF3 mediates mitochondrial homeostasis and improves ischemic stroke through regulating the MAPK signaling pathway
Source: Front Mol Neurosci. 2025 Jun 20;18:1554802. doi: 10.3389/fnmol.2025.1554802 (PMC12226594; doi:10.3389/fnmol.2025.1554802)
Supplement: Supplementary file 1 [file Data_Sheet_1.docx]

**
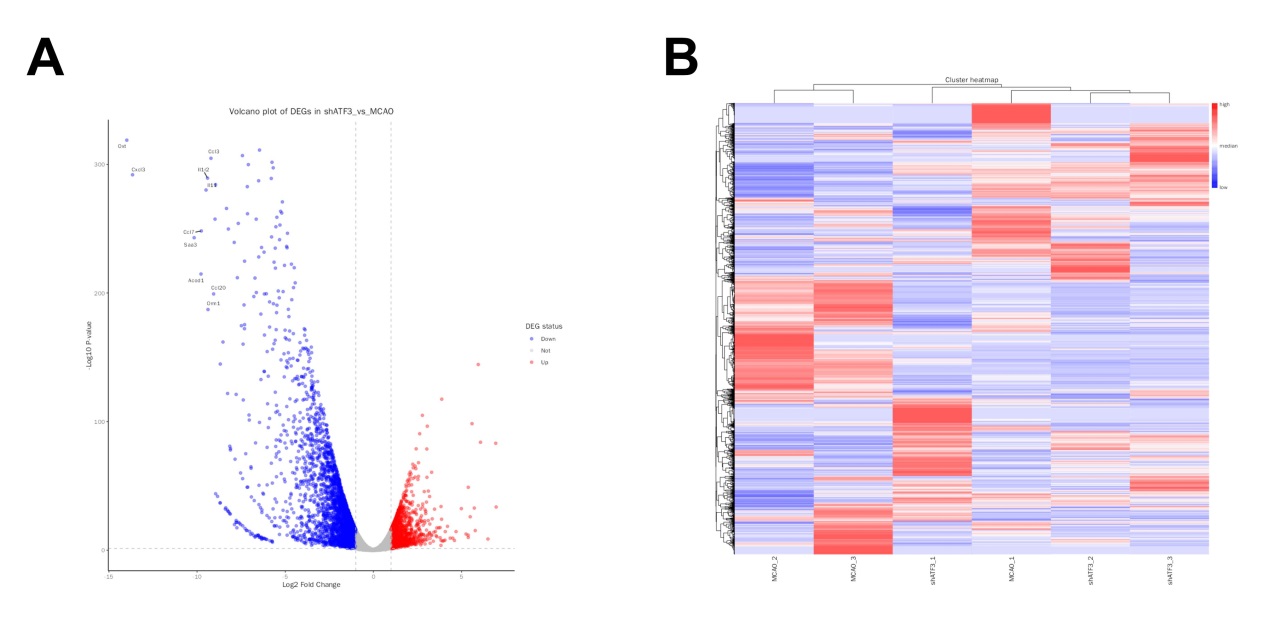
**

**Supplementary Fig 1** Identification of DEGs between MCAO and MCAO+lv-ATF3 groups. A, Volcano plots display a total of 4517 DEGs. Red plots represent upregulated genes and blue plots represent downregulated genes. B, Cluster analysis for 4517 DEGs.

**Supplementary Table 1** Primer sequences for si-NC and si-ATF3

| **Primers** | **Sequences (5’-3’)** |
| --- | --- |
| si-NC-F | UUCUCCGAACGUGUCACGUTT |
| si-NC-R | ACGUGACACGUUCGGAGAATT |
| si-ATF3-1-F | UAUCUGUUGGAUAAAGAGGUU |
| si-ATF3-1-R | CCUCUUUAUCCAACAGAUAAA |
| si-ATF3-2-F | UUAUCUGUUGGAUAAAGAGGU |
| si-ATF3-2-R | CUCUUUAUCCAACAGAUAAAA |
| si-ATF3-3-F | UUUUAUCUGUUGGAUAAAGAG |
| si-ATF3-3-R | CUUUAUCCAACAGAUAAAAGA |

**Supplementary Table 2** The top 15 upregulated genes and top 15 downregulated genes between MCAO and MCAO+lv-ATF3 rats

| **Name** | **log2FoldChange** | **pval** | **up/down** |
| --- | --- | --- | --- |
| Zfp938 | 6.960559003 | 3.6711E-05 | Up |
| LOC134480490 | 6.936859567 | 1.33562E-16 | Up |
| Defb1 | 6.494125248 | 0.000203815 | Up |
| Epyc | 6.073063432 | 0.000349744 | Up |
| Tph1 | 5.945601797 | 0.004471824 | Up |
| LOC134480491 | 5.773917968 | 4.70992E-16 | Up |
| LOC134486359 | 5.722218938 | 1.08959E-05 | Up |
| LOC120097339 | 5.592097951 | 4.93954E-26 | Up |
| Lipm | 5.490064892 | 1.37542E-38 | Up |
| Pax7 | 5.379187811 | 0.001495908 | Up |
| Smr3b | 5.378031016 | 7.76222E-60 | Up |
| LOC134480516 | 5.371713277 | 3.70815E-05 | Up |
| Pou4f2 | 5.251182228 | 4.02604E-59 | Up |
| LOC134480478 | 4.987178475 | 9.4329E-86 | Up |
| Gngt1 | 4.685452707 | 0.001352589 | Up |
| Oxt | -13.9855043 | 1.5937E-319 | Down |
| Cxcl3 | -13.66029706 | 1.2624E-292 | Down |
| Saa3 | -10.16521067 | 1.0772E-243 | Down |
| Acod1 | -9.77792629 | 1.7328E-215 | Down |
| Ccl7 | -9.758876305 | 5.7722E-249 | Down |
| Il11 | -9.491852076 | 8.9212E-281 | Down |
| Il1r2 | -9.404373861 | 5.2731E-290 | Down |
| Orm1 | -9.379446443 | 7.3935E-188 | Down |
| Ccl3 | -9.212213374 | 1.8435E-305 | Down |
| Ccl20 | -9.062617103 | 5.6531E-200 | Down |
| Cebpb | -8.982334223 | 3.7849E-258 | Down |
| Clec4e | -8.951007441 | 5.9032E-285 | Down |
| Neurl3 | -8.945563704 | 1.26502E-44 | Down |
| Il12b | -8.837587286 | 1.42392E-42 | Down |
| Sp9 | -8.696732043 | 1.62169E-37 | Down |
